# A Meta-Analysis of the Effects of the 5-Hydroxytryptamine Transporter Gene-Linked Promoter Region Polymorphism on Susceptibility to Lifelong Premature Ejaculation

**DOI:** 10.1371/journal.pone.0054994

**Published:** 2013-01-30

**Authors:** Lijie Zhu, Yuanyuan Mi, Xiaoming You, Sheng Wu, Hongbao Shao, Feng Dai, Tao Peng, Feng Qin, Ninghan Feng

**Affiliations:** 1 Department of Urology, Third Affiliated Hospital of Nantong University, Wuxi, Jiangsu, China; 2 Department of Urology, First Affiliated Hospital of Nanjing Medical University, Nanjing, Jiangsu, China; Institut Jacques Monod, France

## Abstract

**Objective:**

Premature ejaculation (PE) has been reported as the most common male sexual dysfunction with global prevalence rates estimated at approximately 30%. The neurobiogenesis of ejaculation is very complex and involves the serotoninergic (5-hydroxytryptamine, 5-HT) system. Recently, genetic polymorphisms located on SLC6A4 gene codifying for 5-HT transporter (5-HTT), the major regulator of serotonic neurotransmission, have been linked with the pathogenesis and risk of PE. Apparently studies of this type of polymorphism in PE have show conflicting results.

**Methods:**

A meta-analysis was performed that are available in relation with 5-HTT gene-linked promoter region (*5-HTTLPR*) polymorphism and the risk of lifelong PE (LPE) in men to clarify this relationship. We searched Pubmed and Embase (last search updated on Aug 2012) using ‘premature ejaculation’, ‘polymorphism or variant’, ‘genotype’, ‘ejaculatory function’, and ‘rapid ejaculation’ as keywords and reference lists of studies corresponded to the inclusion criteria for meta-analysis. These studies involved the total number of 481 LPE men and 466 health control men subjects. Odds ratio (OR) and 95% confidence intervals (CIs) were used to evaluate this relationship.

**Results:**

In the overall analysis, significant associations between LPE risk and 5-HTTLPR polymorphism were found (L-allele vs. S-allele OR = 0.86, 95% CI = 0.79–0.95, *P* = 0.002; LL vs. SS: OR = 0.80, 95% CI = 0.68–0.95, *P* = 0.009; LS vs. SS: OR = 0.85, 95% CI = 0.76–0.97, *P* = 0.012 and LL+LS vs. SS: OR = 0.88, 95% CI = 0.81–0.95, *P* = 0.002). Moreover, in subgroup analysis based on ethnicity, similar significant associations were detected. The Egger’s test did not reveal presence of a publication bias.

**Conclusions:**

Our investigations demonstrate that *5-HTTLPR* (L>S) polymorphism might protect men against LPE risk. Further studies based on larger sample size and gene-environment interactions should be conducted the role of *5-HTTLPR* polymorphism and LPE risk.

## Introduction

Premature ejaculation (PE) is one of the most common male sexual complaints [1]–[2]. In addition to adversely influencing sexual relationships, PE significantly impacts the emotional well being and overall quality of life of both men and their partners [3]. In clinical practice, PE is generally distinguished as primary (lifelong), occurring and persisting from the first sexual intercourse and secondary (acquired), occurring after a period of normal control of ejaculatory function [4].

Lifelong PE (LPE) is defined as a male sexual dysfunction characterized by ejaculation that always or nearly always occurs prior to or within about 1 minute of vaginal penetration, the inability to delay ejaculation on all or nearly all vaginal penetrations, and with negative personal consequences, such as distress, bother, frustration, and/or the avoidance of sexual intimacy [5].

The complete etiology of PE is largely unknown. PE has historically been considered a psychological disorder. However, besides psychological, environmental, and contextual aspects, several published articles have focused on neurobiological, endocrine, and genetic causative factors [6]–[8]. Around 30% of the etiology in PE is due to genetic effects [9].

The successful use of selective serotonin reuptake inhibitors (SSRIs) in the treatment of PE indicates that the classical psychological view of PE is no longer tenable as the only possible pathogenetic theory behind PE, and that serotonin (5-hydroxytryptamine, 5-HT) plays a role in the ejaculation process [10]. These findings suggest that the serotonin transporter (5-HTT) gene is a good candidate for genetic studies of PE.

The 5-HTT functioning is moderated by a polymorphism in the 5-HTT promoter region of the serotonin transporter (SERT) gene (SCL6A4), which encodes for the SERT (5-hydroxytryptamine transporter-linked promoter region [5-HTTLPR]) [11], [12]. The *5-HTTLPR* gene has two variant alleles: a short (S) and a long (L) allele. The short allele has 44 base pairs (bps) less than the L allele [13]. The transcriptional activity of the L allele has been reported to be twice as high as the S allele [14]. In vitro studies of the functional effects of this polymorphism show that the long variant is associated with a 3-fold increase in transcriptional activity. It has also been found that levels of serotonin transporter mRNA and serotonin uptake capacity are reduced in lymphoblastoid cell lines that are derived from individuals with one or two copies of the short allele [15].

Several epidemiologic studies have examined associations between *5-HTTLPR* gene polymorphism with potential functional significance and risk of LPE [16]–[21]. However, results have been inconsistent across these studies. Some studies reported this gene polymorphism was a risk factor for LPE, however, different opinions were published that this polymorphism was a protect factor or had no relationship for LPE risk. The objective of our study was to examine associations between *5-HTTLPR* gene polymorphism and risk of LPE in larger samples by meta-analysis.

## Materials and Methods

### Search Strategy

We searched the Pubmed and Embase databases for all articles on the association between *5-HTTLPR* gene polymorphism and LPE risk up to Aug, 2012. The medical subject headings and key words used for search were ‘premature ejaculation’, ‘polymorphism or variant’, ‘genotype’, ‘ejaculatory function’, and ‘rapid ejaculation’. The electronic searching was supplemented by checking reference lists from the identified articles and reviews for additional original reports.

### Inclusion and Exclusion Criteria

Eligible studies had to meet the following criteria: (1) study was designed using the methodology of a case-control study; (2) the association between *5-HTTLPR* gene polymorphism and LPE risk was explored; (3) LPE was operationally defined as the lifelong presence of an intravaginal ejaculation latency time (IELT) of 1 minute or less after vaginal penetration occurring an more than 90% of occasions of sexual intercourse with complaints of inability to delay ejaculation and feelings of frustration about it. The major exclusion criteria were: (1) duplicate data, (2) abstract, comment, review and editorial, (3) no sufficient data were reported and (4) The patients with erectile dysfunction and other sexual problems, such as decreased libido, a history of sexual abuse, chronic prostatitis and infravesical obstruction.

### Data Abstraction

The following items were collected: first author’s last name, year of publication, country of origin, ethnicity, source of control (hospital-based, HB and population-based, PB) and Hardy–Weinberg equilibrium (HWE) of control group, total number and genotype distributions in cases/controls and the L-allele frequency of control groups.

### Statistical Analysis

The strength of the association between the *5-HTTLPR* gene polymorphism and LPE risk was measured by odds ratios (ORs) with 95% confidence intervals (CIs). Pooled ORs were obtained from combination of single studies by allelic contrast (L-allele vs. S-allele), homozygote comparison (LL vs. SS), heterozygote comparison (LS vs. SS), and dominant model (LL+LS vs. SS), respectively. The heterogeneity among the studies was checked by using the chisquare based *Q* statistic and considered statistically significant at *P*<0.05 [22]. When *P*>0.05, the pooled OR of each study was calculated by using the fixed-effects model (the Mantel-Haenszel method, which weights the studies by the inverse of the variance of estimates); otherwise, the random-effects model (the DerSimonian and Laird method) was used [23], [24]. The significance of the pooled OR was determined by the *Z*-test, and *P*<0.05 was considered statistically significant. The departure of frequencies of 5-HTTLPR gene polymorphism from expectation under HWE was assessed by the chi-square test in controls and a *P*<0.05 was considered as significant disequilibrium. Publication bias was diagnosed with Egger’s linear regression method and funnel plot. The *P*-value less than 0.05 in Egger’s linear regression indicated the presence of potential publication bias [25]. All statistical tests for this meta-analysis were performed with Stata software, version 10.0 (STATA Corp., College Station, TX, USA), and all tests were two-sided.

## Results

### Studies Characteristics

**Figure 1:**
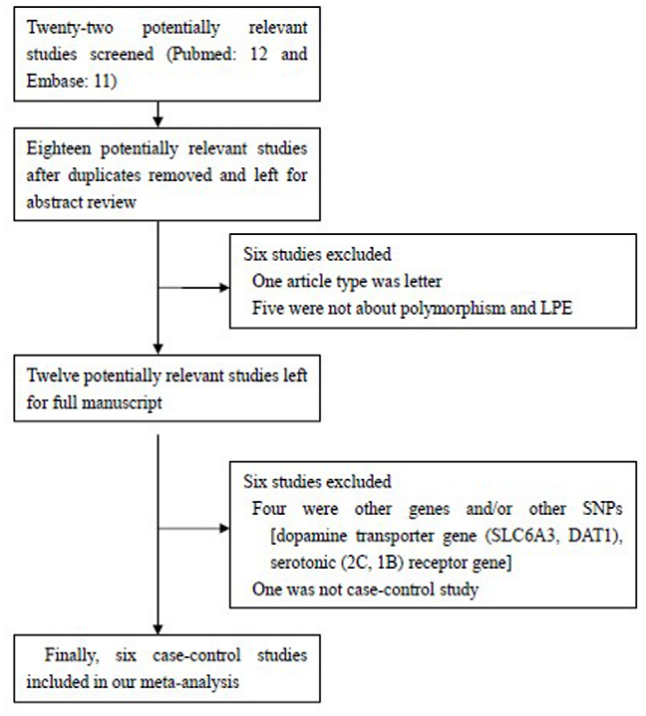
Flow chart of selection of studies and specific reasons for exclusion from the meta-analysis.

A total of 23 articles were achieved by literature search from the Pubmed and Embase, using different combinations of key terms. As shown in **Fig. 1**, seventeen eligible studies were retrieved for abstract review. We excluded six studies: one article type was letter; five were not about polymorphism and LPE. After that, eleven potentially relevant studies were left for full article review. Also six studies were excluded: four were about other genes and/or other SNPs [dopamine transporter gene (SLC6A3, DAT1), serotonic (2C, 1B) receptor gene]; one was not case-control study. In only study by Safarinejad et al. [16], the author further divided the L-allele into L_A_ and L_G_ variants. In our study, we combined these two variants together as L-allele. Finally, six studies with 481 LPE cases and 466 health controls were retrieved based on the search criteria. Characteristics of studies in this meta-analysis are included in **Table 1**. Of these studies, only one was conducted on Asian descendants and five on Caucasian descendants. All studies used the same genotyping method: polymerase chain reaction-based restriction fragment length polymorphism (PCR-RFLP). With the exception of two studies [16], [20], the distribution of genotypes in the controls was consistent with the HWE.

**Table 1:** Characteristics of studies of *5-HTTLPR* polymorphism included in this meta-analysis.

First author/Year	Country	Ethnicity	Source of control	Cases/Controls	Cases	Controls		
					LL/LS/SS	LL/LS/SS	HWE	L%
Safarinejad/2009	Iran	Caucasian	PB	82/82	24/29/29	35/30/17	No	0.61
Janssen/2009	the Netherlands	Caucasian	HB	89/92	27/43/19	27/41/24	Yes	0.52
Luo/2011	China	Asian	HB	119/90	24/34/61	25/31/34	No	0.45
Ozbek/2009	Tukey	Caucasian	PB	69/69	11/21/37	12/37/20	Yes	0.44
Zuccarello/2012	Italy	Caucasian	PB	89/100	22/49/18	33/51/16	Yes	0.59
Jern/2012	Finland	Caucasian	PB	33/33	13/15/5	12/16/5	Yes	0.61

### Meta-analysis

In the overall analysis, significantly decreased associations could be observed between LPE risk and the *5-HTTLPR* gene polymorphism in different genetic models: allelic contrast (OR = 0.86, 95% CI = 0.79–0.95, *P*_heterogeneity_ *= *0.086, *P* = 0.002), homozygote comparison (OR = 0.80, 95% CI = 0.68–0.95, *P*_heterogeneity_ *= *0.240, *P* = 0.009), heterozygote comparison (OR = 0.85, 95% CI = 0.76–0.97, *P*_heterogeneity_ *= *0.047, *P* = 0.012), and dominant genetic model (OR = 0.88, 95% CI = 0.81–0.95, *P*_heterogeneity_ *= *0.020, *P* = 0.002) (**Table 2**).

**Table 2:** Stratified analyses of *5-HTTLPR* gene polymorphism on LPE risk.

Variables	N^a^	Cases/Controls	Allelic contrast	Homozygote comparison	Heterozygote comparison	Dominant genetic model
			OR(95%CI) *P* ^b^/*P*	OR(95%CI) *P* ^b^/*P*	OR(95%CI) *P* ^b^/*P*	OR(95%CI) *P* ^b^/*P*
Total	6	481/466	0.86(0.79–0.95)0.086/0.002	0.80(0.68–0.95)0.240/0.009	0.85(0.76–0.97)0.047/0.012	0.88(0.81–0.95)0.020/0.002
Ethnicity						
Asian	1	119/90	0.64(0.43–0.96)−/0.029	0.54(0.27–1.08)−/0.080	0.61(0.32–1.16)−/0.133	1.47(0.77–2.78)−/0.067
Caucasian	5	362/376	0.88(0.80–0.98)0.086/0.015	0.83(0.70–1.00)0.228/0.046	0.88(0.77–1.00)0.040/0.043	0.90(0.83–0.98)0.024/0.015
Source of control						
HB	2	208/182	0.91(0.78–1.06)0.039/0.229	0.87(0.66–1.15)0.077/0.339	0.93(0.75–1.15)0.083/0.518	0.93(0.80–1.07)0.032/0.294
PB	4	273/284	0.83(0.74–0.93)0.213/0.002	0.76(0.61–0.93)0.395/0.008	0.81(0.70–0.94)0.056/0.006	0.85(0.77–0.94)0.042/0.001

**Figure 2:**
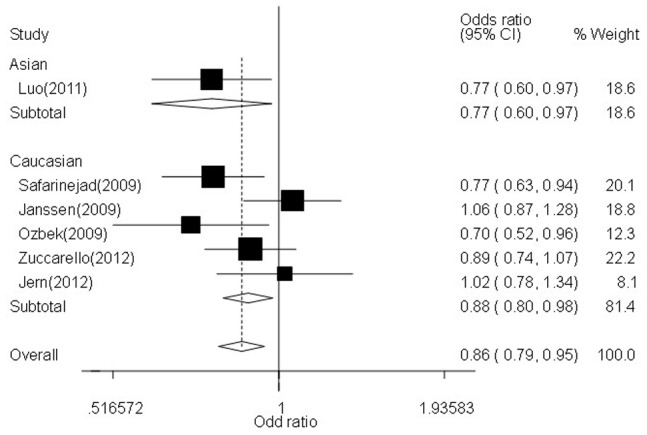
Forest plot of LPE risk associated with the *5-HTTLPR* gene polymorphism (LL vs. SS) by ethnicity subgroup. The squares and horizontal lines correspond to the study-specific OR and 95% CI. The area of the squares reflects the weight (inverse of the variance). The diamond represents the summary OR and 95% CI.

**Figure 3:**
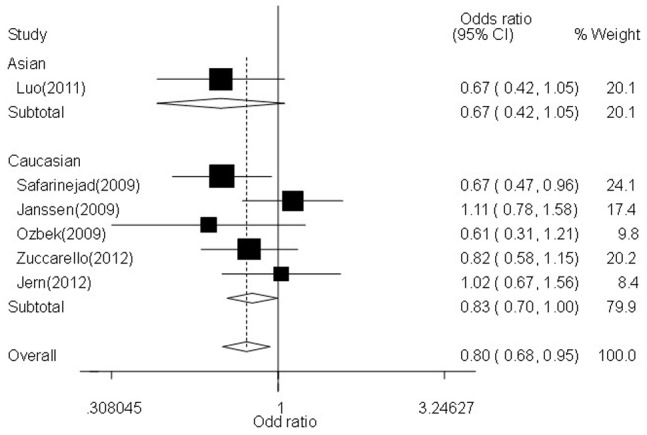
Forest plot of LPE risk associated with the *5-HTTLPR* gene polymorphism (LL +LS vs. SS) by ethnicity subgroup. The squares and horizontal lines correspond to the study-specific OR and 95% CI. The area of the squares reflects the weight (inverse of the variance). The diamond represents the summary OR and 95% CI.

In the stratified analysis by ethnicity subgroup, significantly decreased associations were also found between LPE risk and *5-HTTLPR* gene polymorphism in Caucasians not Asians: allelic contrast (OR = 0.88, 95% CI = 0.80–0.98, *P*_heterogeneity_ *= *0.086, *P* = 0.015), homozygote comparison (OR = 0.83, 95% CI = 0.70–1.00, *P*_heterogeneity_ *= *0.228, *P* = 0.046,**Fig. 2**), heterozygote comparison (OR = 0.88, 95% CI = 0.77–1.00, *P*_heterogeneity_ *= *0.040, *P* = 0.043), and dominant genetic model (OR = 0.90, 95% CI = 0.83–0.98, *P*_heterogeneity_ *= *0.024, *P* = 0.015, **Fig. 3**) (Table 2). Similarly, obvious relationships were detected between LPE risk and *5-HTTLPR* gene polymorphism in PB source of controls groups (**Table 2**).

### Sensitivity Analysis and Publication Bias

Sensitivity analysis was performed to assess the influence of each individual study on the pooled OR by sequential removal of individual studies. Although the genotype distributions in two studies [16], [20] did not follow the HWE, the corresponding overall summary OR was not significantly altered with/without including the study (data not shown). The results suggested that no individual study significantly affected the overall OR dominantly. The Begg’s funnel plot and Egger’s test were performed to assess publication bias. As shown in Table 3, the shapes of the funnel plots did not reveal any obvious asymmetry in any of the comparison models. Then, the Egger’s test was used to provide statistical evidence of funnel plot symmetry. The results still did not suggest any evidence of publication bias (allelic contrast, *t* = −0.74, *P* = 0.501, homozygote comparison, *t* = −0.76, *P* = 0.491; heterozygote comparison, *t* = −1.94, *P* = 0.124, and dominant genetic model, *t* = −2.22, *P* = 0.091, **Table 3**).

**Table 3:** Publication bias tests (Begg’s funnel plot for publication bias test).

Genetic type	Coefficient	Standard error	t	P value	95%CI of intercept
Allelic contrast	−2.437	3.300	−0.74	0.501	(−11.599,6.726)
Homozygote comparison	−1.812	2.394	−0.76	0.491	(−8.457,4.834)
Heterozygote comparison	−3.589	1.846	−1.94	0.124	(−8.715,1.536)
Dominant genetic model	−4.313	1.946	−2.22	0.091	(−9.715,1.089)

## Discussion

The overall goal of meta-analysis is to combine the results of previous studies to arrive at summary conclusions about a body of research. It is most useful in summarizing prior research when individual studies are small, and when they are individually too small to yield a valid conclusion. To the best of our knowledge, this is the first meta-analysis to explore the association between *5-HTTLPR* gene polymorphism and LPE risk, involving about 481 LPE individuals and 466 healthy controls. The main finding of this study is that LL genotype and/or L-allele in *5-HTTLPR* gene polymorphism could protect individuals against LPE risk in Caucasian populations.

While our study provided convincing evidence that this polymorphism played a part in ejaculatory function in humans, it only explains a fraction of the etiological variance in LPE, because the neurobiogenesis of ejaculation is very complex [26], [27]. Waldinger et al. first postulated that the persistent short IELT in men with PE was associated with diminished serotonin neurotransmission, hyperfunction of 5-HT1A receptors, and a hypofunction of 5-HT2C receptors [28]. The function of 5-HT in the central nervous system are controlled by many factors including the 5-HTT, the major regulator of serotonergic neurotransmission, responsible for the active clearance of synaptic 5-HT and then regulation of presynaptic and postsynaptic 5-HT receptor stimulations [8]. The expression rate of 5-HTT is controlled by the 5-HTTLPR polymorphism [29].

The current most popular pharmacotherapeutic approach to treat PE is ‘off-label’ administration of SSRIs (e.g. paroxetine, fluoxetine, sertraline, citalopram, and escitalopram), which are reported to be effective for treating PE [30]–[32]. It was previously suggested that the response to SSRIs is partly under genetic control [33]. It has been shown that the 5-HTTLPR also influences the effectiveness of SSRIs in patients with depression. White L-allele carriers demonstrated higher response rates when treated with various SSRIs antidepressants [34]. Patients with the *5-HTTLPR* SS and/or SL genotype have a higher risk of SSRI non-response [33]. Moreover, previous studies have revealed an association between the occurrence of adverse events during SSRIs treatment and different serotonin transporter genotypes [35], [36]. Evidence suggests that *5-HTTLPR* gene polymorphism moderates the relationships between affective outcomes and major life events [37]. It is known that the S-allele induces lower 5HTT expression and 5HT reuptake activity than the L-allele [38].

Several epidemiological studies have investigated the association between *5-HTTLPR* gene polymorphism and PE/LPE, but the results were inconclusive. Janssen et al. [17] reported that no association was found between this polymorphism and LPE, however, men carried SS or SL genotype had longer ejaculation time that LL genotype. Similarly, Zuccarello et al. [19] also reported that no statistically significant differences were found in the frequency of *5-HTTLPR* gene polymorphism in LPE patients vs. controls. In contrast, two studies [18], [20] suggested the SS genotype was significantly higher in patients with PE than in the controls (*P*<0.01), however, the LS genotype was more prevalent in the control group (*P*<0.01). Our study showed partly similar conclusions with above two studies. However, two included studies [16], [18] have previously been criticized for potential methodological limitations that the populations of both studies were not in HWE. [39]–[41]. While this criticismis obviously potentially valid, however, it should be pointed out that deviation from HWE is not necessarily indicative of sampling inadequacy or neglect, since neither allele nor genotype frequencies are free from disturbing influences in nature [42]. For example, non-randommating, migration and natural selection will distort allele (and thus genotype) frequencies in populations [43].

We first analyzed the overall association between *5-HTTLPR* gene polymorphism and LPE: LL genotype or L-allele was a protect factor for LPE. Because of the coupled and complementary association between L-allele and S-allele, in contrast, SS genotype and/or S-allele were risk factors of LPE. Then, we analyzed the association stratified analysis by ethnicity, the similar conclusion was found in Asians not Caucasians, which can be explained that the prevalence of PE is different in distinct geographic locations and ethnicities. The Global Study of Sexual Attitudes and Behaviors (GSSAB) reported a PE prevalence ranging from 12% in the Middle East to 30% in Southeast Asia among men 40–80 years of age [44]. In a separate study of men between the ages of 18 and 59 years in the United States, the overall prevalence of PE was 21%, which comprised 19% of Caucasian men, 27% of Hispanic men, and 34% of African-American men [45]. Finally, in case-control studies whose controls were from PB, significant association was also found. Our study may help scientists to detect this SNP in *5-HTTLPR* gene in healthy men and to find high-risk group of PE in the future.

Although, we have put considerable efforts and resources into testing possible association between *5-HTTLPR* gene polymorphism and LPE risk, there are still some limitations inherited from the published studies. First, although we collected all the eligible studies, the sample size of the included studies was not large enough, which could increase the likehood of type I and type II errors. Second, gene-gene and gene-environment interactions were not analyzed. It is possible that specific environmental and lifestyle factors may alter those associations between gene polymorphism and LPE. Third, the distribution of genotypes in the controls of two studies [16], [20] was not consistent with the HWE, which would affect the power of our conclusions. In spite of these, our meta-analysis also had four advantages. First, publication bias was not detected in all genetic models, suggesting that the results were relatively stable and powerful. Second, the quality of case–control studies included in the current meta-analysis was satisfactory based on our selection criteria. Third, the control groups were all healthy men. Fourth, the included studies were published from 2009 to 2012 and quite neoteric.

### Conclusions

In summary, in the present, our meta-analysis showed the evidence that *5-HTTLPR* gene polymorphism was associated with significantly decreased risk for LPE risk in Caucasians. Therefore, further well designed large studies, particularly referring to gene–gene and gene–environment interactions are warranted. These future studies should lead to better and comprehensive understanding of the association between the *5-HTTLPR* gene polymorphism and development of LPE.
